# Efficiently-cooled plasmonic amorphous silicon solar cells integrated with a nano-coated heat-pipe plate

**DOI:** 10.1038/srep24972

**Published:** 2016-04-26

**Authors:** Yinan Zhang, Yanping Du, Clifford Shum, Boyuan Cai, Nam Cao Hoai Le, Xi Chen, Benjamin Duck, Christopher Fell, Yonggang Zhu, Min Gu

**Affiliations:** 1 Centre for Micro-Photonics, Faculty of Science, Engineering and Technology, Swinburne University of Technology, Hawthorn, VIC 3122, Australia; 2 CSIRO Manufacturing Flagship, Gate 5, Normanby Road, Clayton, VIC 3168, Australia; 3 Melbourne Centre for Nanofabrication, 151 Wellington Road, Clayton, VIC 3168, Australia; 4 School of Science, RMIT University, Melbourne, VIC 3001, Australia

## Abstract

Solar photovoltaics (PV) are emerging as a major alternative energy source. The cost of PV electricity depends on the efficiency of conversion of light to electricity. Despite of steady growth in the efficiency for several decades, little has been achieved to reduce the impact of real-world operating temperatures on this efficiency. Here we demonstrate a highly efficient cooling solution to the recently emerging high performance plasmonic solar cell technology by integrating an advanced nano-coated heat-pipe plate. This thermal cooling technology, efficient for both summer and winter time, demonstrates the heat transportation capability up to ten times higher than those of the metal plate and the conventional wickless heat-pipe plates. The reduction in temperature rise of the plasmonic solar cells operating under one sun condition can be as high as 46%, leading to an approximate 56% recovery in efficiency, which dramatically increases the energy yield of the plasmonic solar cells. This newly-developed, thermally-managed plasmonic solar cell device significantly extends the application scope of PV for highly efficient solar energy conversion.

The future viability of energy supply depends on a paradigm shift away from pollution-generating, non-renewable fossil fuels to renewable sources. Solar photovoltaics (PV) are emerging as a major alternative energy source^1^,^2^. However the cost of PV remains an important factor in the rate of expansion of this technology. The cost of PV electricity on a per kWh basis depends on the efficiency of conversion of light to electricity. Despite of steady growth in the efficiency for several decades, little has been achieved to reduce the impact of real-world operating temperatures on this efficiency, and consequently on PV output^3^,^4^,^5^,^6^,^7^. For example, for every 1 K temperature increase of the crystalline Si solar cells, the efficiency would decline by approximately 0.5%^8^. For a PV module operating at up to 60 °C in the field, the long term output may be reduced by 15%, which is equivalent to a loss of around 9 MWh for a typical household with a 2 kW system for 20 years.

The recently-emerged plasmonic solar cells employing both the strong scattering and near-field light concentration of the metal nanoparticles/nanostructures have shown great promise in transforming the PV industry as a cost-effective solution^9^,^10^,^11^,^12^,^13^,^14^,^15^,^16^,^17^,^18^,^19^,^20^,^21^,^22^. The plasmonic effect allows for significantly reducing the thickness of the active layers of the solar cells through introducing a minor amount of metallic nanomaterials while maintaining the high efficiency. A recent breakthrough has been achieved by using lumpy plasmonic Ag nanoparticles on the state-of-the-art amorphous Si (a-Si) thin film solar cells, with a record efficiency above 10%^22^. However, the efficiency gain specified at the standard test condition (STC) can be easily reduced or even completely offset by the thermal effect when the solar cells are operating in the real condition. To fully appreciate the benefits of the plasmonic-induced efficiency enhancement, a highly efficient thermal management strategy is pressingly demanded.

A variety of heat management technologies have been proposed and demonstrated, including active air ducts cooling^23^, water cooling^24^, heat-pipe-based cooling^25^,^26^ and passively thermal radiation^27^,^28^. Among these methods, the heat-pipe plate is a device that can transfer a large amount of heat from hot spots to places with a considerable distance by the liquid-vapour phase change of the working fluid inside the plate^29^,^30^,^31^. The effective thermal conductivity of such a plate can approach as high as 100 kW/m^*^K in a long distance^32^, making it an ideal option as a thermal management system for solar cells. Furthermore, the heat-pipe plate has no moving parts and it is power-independent, long-lasting and inherently integratable with solar panels.

We demonstrate a highly-efficient and thermally-stable plasmonic thin film a-Si solar cell integrated with an advanced nano-coated heat-pipe plate. The heat transportation capability of the developed heat-pipe plate is up to ten times higher than that of the metal plate and the conventional wickless heat-pipe plates. This significant feature originates from the internal microgrooves attached with nano-coated compressed metal foams, with a wicking property of 84% higher than the current porous metals. The resultant reduction in temperature rise for the plasmonic solar cells can be as high as 46%, leading to a significant efficiency recovery of the temperature-induced reduction.

## Integrated plasmonic solar cell device

**Figure 1:**
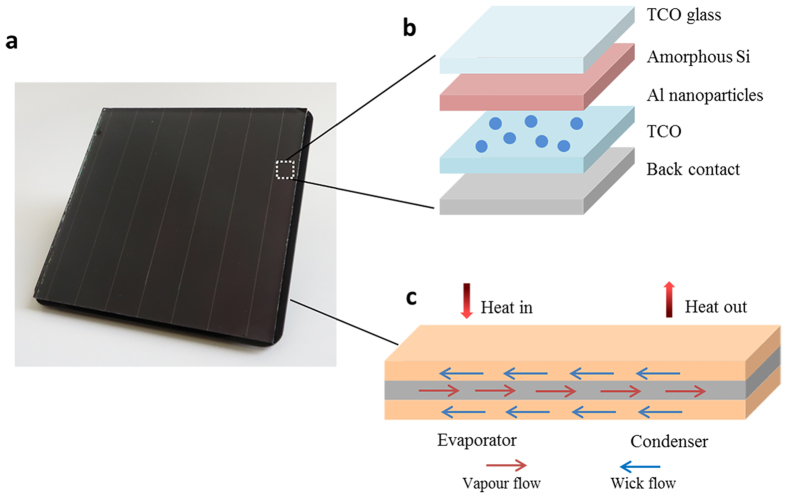
Heat-pipe plate managed plasmonic solar cells. (**a**) A photo of the plasmonic amorphous Si solar panels (10 cm * 10 cm) integrated with the heat-pipe plate. (**b**) Schematic diagram of the plasmonic amorphous Si solar cells. (**c**) Working mechanism of the heat-pipe plate.

Figure 1a shows a photo of the plasmonic solar cell integrated with the newly developed heat-pipe plate through the interface material (i.e. thermal pad layer). The plasmonic solar cell consists of TCO glass/a-Si/Al nanoparticles/TCO/Al reflector, as schematically shown in Fig. 1b. The thin heat-pipe plate (Fig. 1c) is a sealed, hollow copper plate partially filled with deionized (DI) water as the working fluid. The heat generated in the solar cells is firstly conducted to the evaporation end of the heat-pipe plate. The vapour generated from the evaporation of water will flow to the condenser section, bringing along a large amount of latent heat which is dissipated to the ambient by means of natural convection and thermal radiation. As a result, the vapour condenses to liquid, which autonomously returns to the evaporation end by capillary force generated from the microcapillaries and nano-coated wicking materials. Through integration with the heat-pipe plate, the energy conversion efficiency of solar cells can be significantly recovered, demonstrating the value of the integrated device for economic benefits.

## Performance of the nano-coated heat-pipe plate

**Figure 2:**
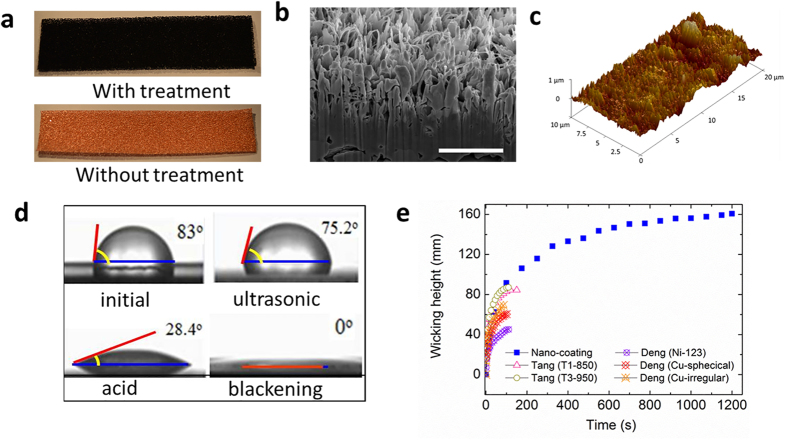
Performance of the nano-coated compressed metal foam. (**a**) Photos of the metal foams with and without treatment. (**b**) SEM image of the cross-section of the treated metal foams with a cut-out by a focused ion beam (FIB) to expose the structure (scale bar: 2 μm). (**c**) AFM image of the surface of the treated metal foams. (**d**) Contact angle of the degassed water on the in-process metal foams. (**e**) Wicking performance of the nano-coated metal foams in comparison with the published data^33^,^34^.

To address the applications to solar cells for cooling and increasing energy yield, the heat-pipe plate should be thin for easy integration and low cost for commercial viability. This requires the wicking structures to generate sufficient capillary force for long distance liquid wicking (up to 1 m). We designed the thin plate format with inside walls fabricated with microcapillaries and attached with compressed copper foams with CuO nano-coating. The commercially available copper foams were compressed to improve the capillary force due to the reduced pore size of the porous media. The geometric parameters of the compressed copper foams are listed in Supplementary Table 1. The CuO nano-coating layer on the surface of the copper foams was achieved through ultrasonic washing, acid washing and blackening process. Figure 2a shows the photos of the nano-coated metal foams in comparison with that without nano-coating. The CuO nano-layer has an average roughness of 104 nm, as demonstrated by the SEM (Fig. 2b) and AFM (Fig. 2c) images. The SEM image of the treated metal foams, compared with that without the treatment is shown in Supplementary Fig. 1.

The improved micro-roughness of the fibrotic nano-layer leads to a significant wettability increase of the DI water on the copper foams, demonstrated by a largely reduced contact angle. The original metal foams surface was hydrophobic and thus the contact angle was as high as 83° (Fig. 2d). The wettability was significantly improved by ultrasonic washing and acid treatment with the measured contact angle reduced to 75.2° and 28.4°, respectively. The blackening process turned the surface of metal foams to be super hydrophilic, resulting in a contact angle close to almost 0°. As a result, the wicking behaviour in the current porous material is significantly improved. This improvement is quantified through a wicking experiment where the wicking speed and height are measured when a strip of the porous material is allowed to contact with water. The measured maximum wicking height is 162 mm, which is 84% higher than the published results under the same testing conditions (Fig. 2e). It is clear that the treated metal foams have much better wicking properties. The better wicking performance allows the heat-pipe plate to transport liquid back efficiently, which is a crucial requirement for the efficient heat transfer by the heat-pipe plate.

**Figure 3:**
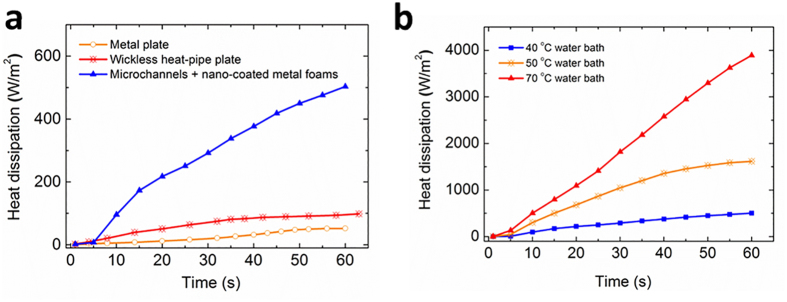
Performance of the nano-coated heat-pipe plate. (**a**) Heat transport comparison between the nano-coated heat-pipe plate, the metal plate and the conventional wickless heat-pipe plate at a test temperature of 40 °C. (**b**) Heat removal capacity of the nano-coated heat-pipe plate in the applicable temperature range.

Before the integration with solar cells, the heat removal performance of the heat-pipe plates was experimentally investigated. One end of the heat-pipe plate was immersed in the water bath with a controlled temperature in the range of 40~70 °C. The saturated temperature of the working fluid (DI water) was below 40 °C for the given vacuum pressure of the device. Figure 3a compares the heat removal rate of the heat-pipe plate under a temperature difference of 20 °C (i.e., 40 °C of water bath temperature over the ambient temperature of 20 °C) with those of the conventional heat-pipe and metal plates as the benchmark. Conventional heat pipe refers to the one that wicking happens in channels or grooves of different shapes while the metal plates we used is a copper plate with a thermal conductivity at around 380 W/m^*^K. The current design is much more superior to other designs as the heat dissipation is approximately 10 and 6 times higher than those of the metal plate and a conventional wickless heat-pipe plate, respectively. Figure 3b demonstrates the heat removal capacity of the developed heat-pipe plate in an applicable operating temperature range (40~70 °C or 20~50 °C over the ambient temperature), which indicates a much more pronounced heat removal at larger temperature differences.

## Thermal performance of the integrated device

**Figure 4:**
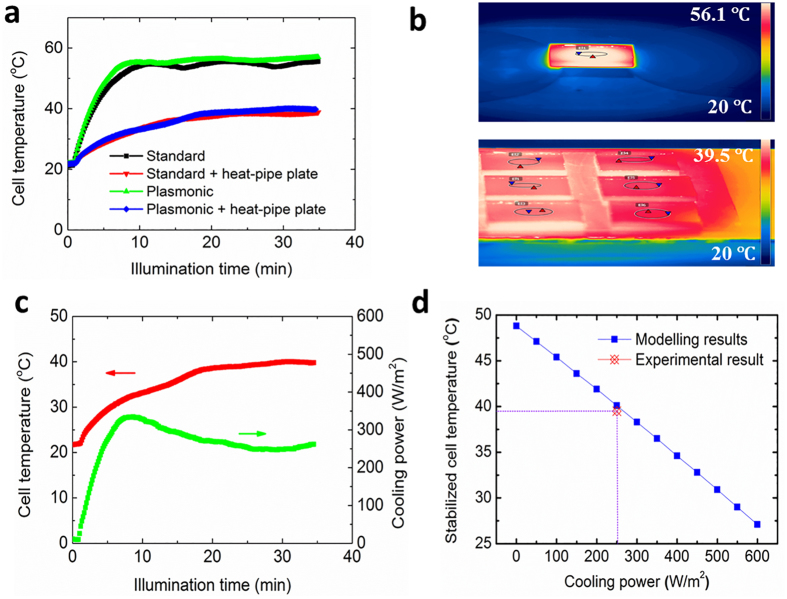
Thermal performance of the integrated device. (**a**) Temperature rise of the solar cells under one sun illumination at 20 °C ambient temperature for different device configurations: standard, standard + heat-pipe plate, plasmonic, plasmonic + heat-pipe plate. (**b**) Stabilized temperature distributions for the plasmonic solar cells with (bottom image) and without (top image) the integrated heat-pipe plate (the temperature values of the scale bar are averaged across the circle areas). (**c**) Cooling power variation of the device during the illumination. (**d**) Relation of the cooling power and the stabilized cell temperature.

Figure 4a shows the temperature rise of the plasmonic and standard a-Si solar cells integrated with the developed heat-pipe plate under the solar simulator illumination. It can be seen that the operating temperature of the cells was dramatically reduced from 56.1 °C to 39.5 °C due to the cooling by the heat-pipe plate, representing an approximate 46% temperature-rise reduction under the ambient temperature of 20 °C. However, the temperature stabilization of the integrated device took longer time than that without heat-pipe thermal management, due to the enlarged heat capacity considering the mass of the heat-pipe plate. The field test results of the 10 cm × 10 cm thin-film solar panels show an average temperature-rise reduction of 40% and 47% for both the summer and winter time, demonstrating the highly effective cooling of the nano-coated heat-pipe plate in practical application (Supplementary Fig. 2). It is interesting to note that the plasmonic cell temperature is slightly higher (1–2 °C) than that of the standard cells indicating that the integrated nanoparticles are weakly light-absorbing, which is favourable to the solar cell performance. To confirm this, we calculated the normalized absorption cross-section of the 200 nm Al nanoparticle across the entire solar spectrum range from 0.3 μm to 2.5 μm by the finite different time domain (FDTD) method. The low value of the normalized absorption cross-section (Supplementary Fig. 3), particularly at wavelengths above 1000 nm, in combination with the low surface coverage density (10%) of the Al nanoparticles suggest that a negligible amount of light is absorbed by the nanoparticles and converted to heat.

Figure 4b shows the infrared imaging of the surface temperature of the plasmonic solar cells without and with the heat-pipe plate, respectively. The temperature distribution of the solar cell without the heat-pipe plate shows a radial pattern with a higher temperature at the central area. The temperature gradient is as high as 3 °C/cm. In comparison, the solar cells integrated with the heat-pipe plate present a much more uniform temperature distribution across the cell area. This indicates that the thermal energy produced in the cells prefers to transport vertically to the heatpipe plate instead of horizontally transferring to the outside air space. The uniform heat distribution is especially favourable for large-scale high performance solar modules with a series of individual solar cells interconnected, since the non-uniformity could lead to the current mismatch between the individual cells thereby a performance decline.

Based on the experimental data, the cooling power by the heat-pipe plate is evaluated using the energy balance method^35^. The results indicate that the cooling power of the heat-pipe plate increases simultaneously with the temperature rise of the solar cells, which is stabilized as 250–260 W/m^2^ with a steady cell temperature of approximately 39.5 °C (Fig. 4c). The relationship of the stabilized cooling power and cell temperature is also calculated (Fig. 4d). With the cooling power increased, the solar cell temperature is reduced accordingly. For a cooling power of above 600 W/m^2^, the cell temperature is expected to be maintained close to 25 °C. For the current integration with a cooling power of 250 W/m^2^, the calculated panel temperature is 40.1 °C, showing a good agreement with the experimental result (39.5 °C).

For the integrated device, the heat removal capacity is significantly affected by the integration-induced thermal resistance and thermal dissipation strategies. We performed the thermal resistance analysis of the current integration method, in comparison with the ideal case and that without the thermal pad layer (Supplementary Fig. 4). The results indicate the current integration method is highly effective, with the cooling power approaching to the ideal case. To further improve the cooling effect, advanced thermal dissipation strategies or energy utilisation strategies are needed to accelerate the heat dissipation.

## Electrical performance of the integrated device

**Figure 5:**
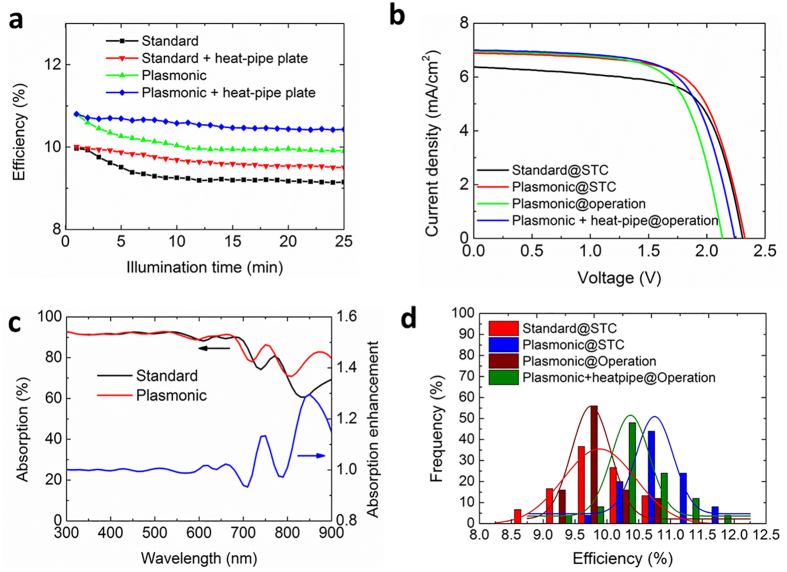
Electrical performance of the devices. (**a**) The energy conversion efficiency decline of the standard and plasmonic cells with and without the heat-pipe plate while the cells are illuminated with one sun. (**b**) The current density-voltage (*J-V*) curves of the devices at different configurations: standard and plasmonic solar cells at standard test conditions (STC), plasmonic solar cell operating under one sun without and with the heat-pipe plate integrated. (**c**) The absorption of the standard and plasmonic cells. (**d**) The efficiency statistical distribution for the devices under different configurations.

Figure 5a shows the energy conversion efficiency degradation of both the plasmonic and the standard solar cells when they are illuminated under the solar simulator, starting from 25 °C. Figure 5b gives the corresponding current density-voltage (J-V) curves of the standard solar cells (black) and the plasmonic solar cells (red) specified under STC and plasmonic solar cells operating under one sun with (blue) and without (green) the heat-pipe plate. The detailed electrical parameters are shown in Supplementary Table 2. As seen in the figure, the initial efficiency of the solar cells is increased from 10% to 10.8% due to the short-circuit current density increase (from 6.4 mA/cm^2^ to 6.9 mA/cm^2^). This is a result of the absorption enhancement (Fig. 5c) of the solar cells by the nanoparticle light trapping. Clearly, the absorption at the wavelengths above the wavelength of 600 nm is strongly enhanced, with an enhancement up to 30% at around wavelength 850 nm. This is induced by both the scattering and the near-field light concentration of the Al nanoparticles (Supplementary Fig. 3). The SEM image and the UV-Vis spectrum of the nanoparticles are shown in Supplementary Fig. 5. The efficiency of the plasmonic cell declines to around 9.9% after approximately 10 mins illumination from 10.8% as a result of the temperature increase. It can be seen the efficiency gain by the nanoparticles is completely offset. Once the plasmonic cell is integrated with heat-pipe plate, more than 50% of the temperature-induced efficiency loss is recovered, with the efficiency stabilized at around 10.4%. The major factor that contributes to the temperature-induced efficiency reduction and recovery is the open circuit voltage *V*_*oc*_. The voltage reduces as the solar cell temperature increases. This is attributed to the bandgap reduction of the semiconductor material while the temperature increases leading to an increase in the intrinsic carrier concentration. This in turn increases the dark saturation current thereby a *V*_*oc*_ decrease. The voltage is recovered from 2.13 V to around 2.24 V when the solar cell is integrated with the heat-pipe plate. The efficiency reduction and recovery agree well with the predication suggested by the temperature coefficients of the solar cells shown in Supplementary Fig. 6.

To demonstrate the repeatability and reliability of the cooling of the heat-pipe plate, 36 solar panels were used to integrate on the heat-pipe plates to perform a statistical experiment. Figure 5d gives the statistical distribution of the cell efficiency under different conditions. It is obvious the efficiency enhancement by the plasmonic nanoparticles is completely offset when the cells are operating at elevated temperatures and the integration of heat-pipe plate could lead to a recovery of the efficiency loss of more than 50%.

Despite of the attractive improvements in both thermal and electrical performance, it is crucial to analyze the techno-economics of the developed system. An estimated analysis was performed based on investment and gain of a 1 m^2^ solar cell for inland area of Australia. The results show that the developed system becomes economical within approximately 7 years. With increased time of using it, for example, in 20 years, the gain on investment of 1 m^2^ solar cell could reach up to 440 AUD, corresponding to an annual gain of 22 AUD/m^2^.

In conclusion, we have proposed and demonstrated a highly efficient cooling solution for the plasmonic thin-film a-Si solar cells integrated with the low-cost and earth-abundant Al nanoparticles. The cooling solution refers to a novel heat-pipe plate internally structured by the microgrooves in combination with the nano-coated metal foams. Up to 56% of the efficiency loss can be recovered for a plasmonic solar cell that is exposed in a standard solar illumination (AM 1.5 G, 1000 W/m^2^). With active heat dissipation or utilisation strategies applied, the recovery can be further enhanced. This technology could potentially be used in large scale integration with PV cells.

## Methods

### Nano-coated compressed metal foam characterization

The water contact angle measurements were captured by the pocket goniometer PG-3, each deionized water drop was set at 4 μL. Measurements at three locations along of strip were averaged to characterise the contact angle for the particular stage. The wicking height is recorded by the FLIR A6500sc IR camera (or the Basler image processing camera A602fc-2 if the interface can be optically captured) at a minimum frame rate of 0.5 (e.g. 2 frames per second). In order to ensure the metal foam strip is straight during the test, a weight is attached in the bottom. Purified deionized water is used by the simplicity UV ultrapure water system.

### Heat-pipe fabrication and characterization

Completed heat-pipe plate fabrication processes include CNC (Computer Numerical Control) machining of metal plate, clamping, soldering and TIG (Tungsten Inert Gas) welding, followed by the evacuating, degassing and charging. During vacuuming, a turbo molecular pump (Agilent TPS-compact, USA) with a base pressure of 1 × 10^−8 ^mbar was used for lowering the panel pressure. As the metal foam and the internal copper surface keep out gassing, it took approximately one week to reach to the vacuum level of 3 × 10^−5^ mbar. Similar evacuating procedures can be found in ref. 29. The technique reported by Reinarts *et al.* was employed for degassing the DI water before charging the heat-pipe^31^. 200 ml of DI water was filled in a glass flask which was submerged in a heated water tank at 50 °C. A constant vacuum pressure of 90 kPa was applied to the flask to move the dissolved gases from the water to the cold trap. Typically, it takes 30 min for this degassing process. The apparatuses and procedures used for charging the degassed water are shown in the Supplementary Fig. 7. The evaporator end of the heatpipe plate was immersed 10 ~ 30 mm deep in the water contained in the water-bath tank. The infrared camera (MODEL NO SC7000) was used to record the temperature variation during the process of heat transfer through the plate. Then the Rohesenow correlation was adopted to evaluate the heat removal rate^36^.

### Nanoparticle synthesis, characterization and integration

A thin layer (25 nm) of Al film was first evaporated on the surface of the NaCl powder filled in a petri dish via the electron beam evaporator (AXXIS, Kurt J. Lesker) at a deposition rate of 2 Ǻ/s and a vacuum pressure of 1 * 10^−6^ Torr. Then the Al coated NaCl powder was annealed at 400 °C for one hour in a furnace with the nitrogen gas. Due to the surface tension, the nanoparticles coalesce together to form Al island. After this, the powder was dissolved in the DI water, followed by a centrifuge process at a speed of 600 rpm. This allows for the separation of the Al nanoparticles from the NaCl solution. UV-Vis spectrum of the Al nanoparticle solution was performed using a spectrophotometer (Lambda 1050, PerkinElmer). An airgun (ICM4502, Iwata Airbrush) was employed to spray-coat the Al nanoparticles at the back side of the a-Si layer. Through carefully controlling the amount of the nanomaterial solution, approximately 10% coverage density in an area up to 10 cm * 10 cm can be achieved, which is the optimum condition suggested by the finite difference time domain (FDTD) calculation. During the spray coating, the solar cells were heated to around 150 °C on a hotplate, making the solution sprayed on solar cells evaporating quickly thereby leaving nanoparticles evenly on solar cell surface.

### Solar cell fabrication and characterization

The a-Si solar cells were fabricated by the plasmon enhanced chemical vapour deposition (PECVD) system with a structure consisting of glass/TCO/a-Si/Al nanoparticles/TCO/Al reflector. The integrated device was illuminated using the solar simulator (Oriel Sol 3A^TM^ class AAA, model 94023A) with the AM 1.5 G solar spectrum at the room temperature 20 °C. An infrared camera (FLIR ATS-L0106) was used to monitor and record the temperature of the device when the light starts shining on the cells. At the same time, the current-voltage (*I-V*) sweep was performed during the temperature rise of the cells. The absorption measurement of the solar cells was performed using the spectrophotometer (Lambda 1050, PerkinElmer) with an integration sphere.

### Integration of solar cells to a heat-pipe plate

A thin (0.5 mm) layer of thermal pad (TGX, t-global), made from silicone (65%) and aluminium oxide (35%), with a thermal conductivity of 12 W/m*K, was chosen to fill the gap between the solar cells and the heat-pipe plate to ensure the heat flow goes through the joint face efficiently. A slight pressure was applied on the cell surface in order to thoroughly fill up the gap.

## Additional Information

**How to cite this article**: Zhang, Y. *et al.* Efficiently-cooled plasmonic amorphous silicon solar cells integrated with a nano-coated heat-pipe plate. *Sci. Rep.* **6**, 24972; doi: 10.1038/srep24972 (2016).

## Supplementary Material

Supplementary Information
