# Supplementary material for: Efficiently-cooled plasmonic amorphous silicon solar cells integrated with a nano-coated heat-pipe plate
Source: Sci Rep. 2016 Apr 26;6:24972. doi: 10.1038/srep24972 (PMC4844969; doi:10.1038/srep24972)
Supplement: Supplementary Information [file srep24972-s1.pdf]

Supplementary information

Efficiently-cooled plasmonic amorphous silicon solar cells integrated with a nano-coated heat-pipe plate

Yinan Zhang[1,*], Yanping Du[2,*], Clifford Shum[2], Boyuan Cai[1], Nam Cao Hoai Le[2], Xi Chen[1], Benjamin Duck[2], Christopher Fell[2], Yonggang Zhu[2, 3] and Min Gu[1, 4]

[1]Centre for Micro-Photonics, Faculty of Science, Engineering and Technology, Swinburne University of Technology, Hawthorn, VIC 3122, Australia

[2]CSIRO Manufacturing Flagship, Gate 5, Normanby Road, Clayton, VIC 3168, Australia

[3]Melbourne Centre for Nanofabrication, 151 Wellington Road, Clayton, VIC 3168, Australia

[4]School of Science, RMIT University, Melbourne, VIC 3001, Australia

[*]These authors contributed equally to this work. Correspondence and requests for materials should be addressed to M.G. (email: min.gu@rmit.edu.au) and Y. Z. (email: yonggang.zhu@csiro.au)

Table 1. Basic parameters of the compressed metal foams. *δ, ε, PPI,* d_f, d_p denote the thickness, porosity, pore density, fibre diameter and pore diameter of the metal foams; *P* represents the equivalent pressure induced by capillary force in the porous sample.

	δ (mm)	*ε*	*PPI*	d_f*(μm)*	d_p*(μm)*	*P*	
						(Pa)	*(mmH_2O)*
Original foams	2.0	0.9	110	40	231	603	60
Compressed foams	0.4	0.5	246	40	103	1352	133
	0.25	0.2	311	40	81	1706	168

Table 2. Electrical parameters of the solar cells under different configurations. E_{ff}: energy conversion efficiency; V_{oc}: open circuit voltage; J_{sc}: short circuit current density; *FF*: fill factor; *T*: temperature; STC: standard test condition.

	Standard @ STC	Plasmonic @ STC	Plasmonic @ operation	Plasmonic + heat-pipe plate @ operation
E_{ff} *(%)*	10	10.8	9.9	10.4
V_{oc} *(V)*	2.31	2.32	2.11	2.27
J_{sc} *(mA/cm^2)*	6.4	6.9	7	6.9
FF (%)	67.8	67.3	67	66
T(^{o}C)	25	25	56.1	39.5

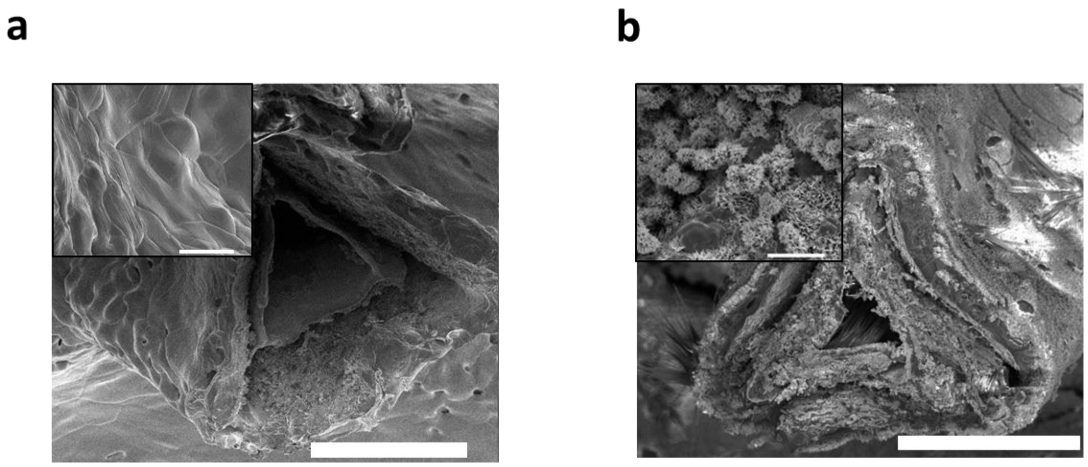

Figure 1. SEM images of the untreated (a) and treated (b) metal foams (scale bar: 40 μm). The inset is the images of the foams at a larger magnification (scale bar: 10 μm)

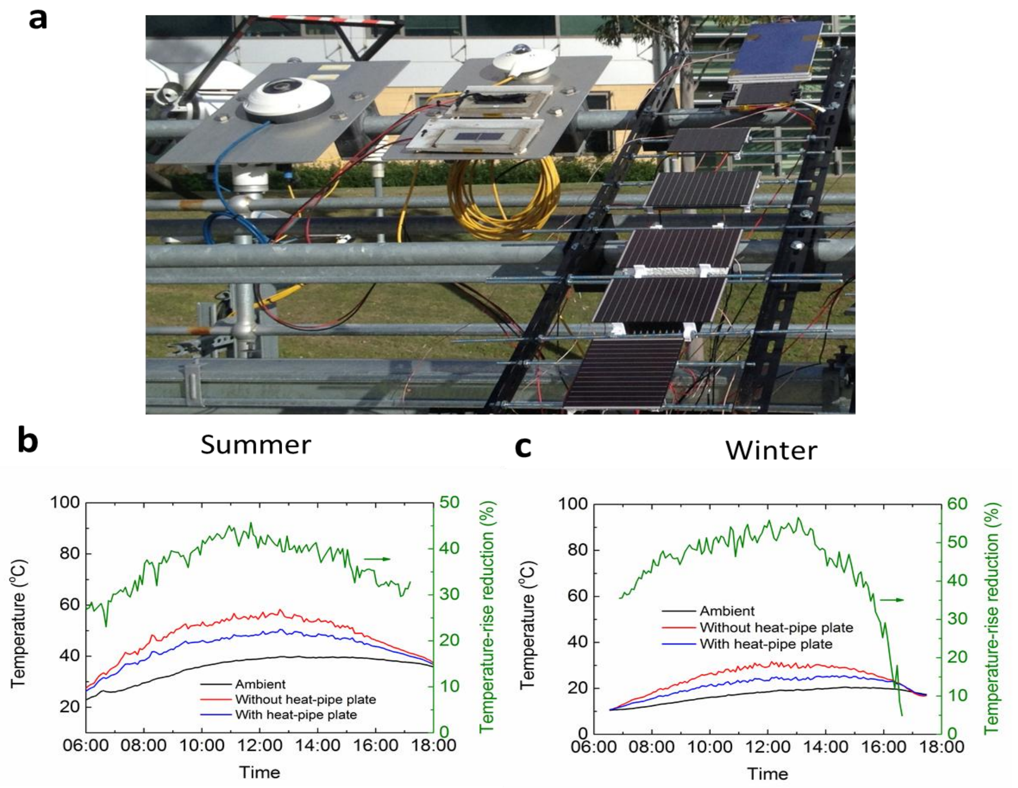

Figure 2. Field test of the 10 cm *10 cm thin-film solar panels integrated with the heat-pipe plate. (a) A photo of the field test set-up with a few 10 cm*10 cm solar panels; (b) Temperature variation of the thin film solar panels in a summer day (20th Nov,

2015); (c) Temperature variation of the thin-film solar panels in a winter day (7th May, 2015). Both sets of data were collected in the outdoor solar cell testing facility at Newcastle, Australia. The average temperature-rise reduction is approximately 40% and 48% for summer and winter tests, respectively.

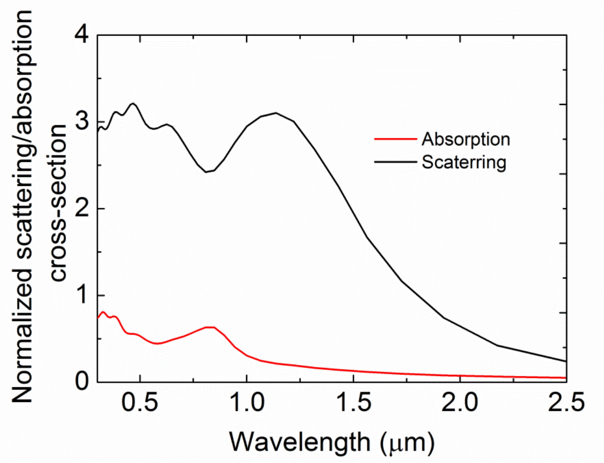

Figure 3. Plasmonic properties of the 200 nm Al nanoparticles. Calculated normalized scattering/absorption cross-sections using the finite difference time domain (FDTD) method.

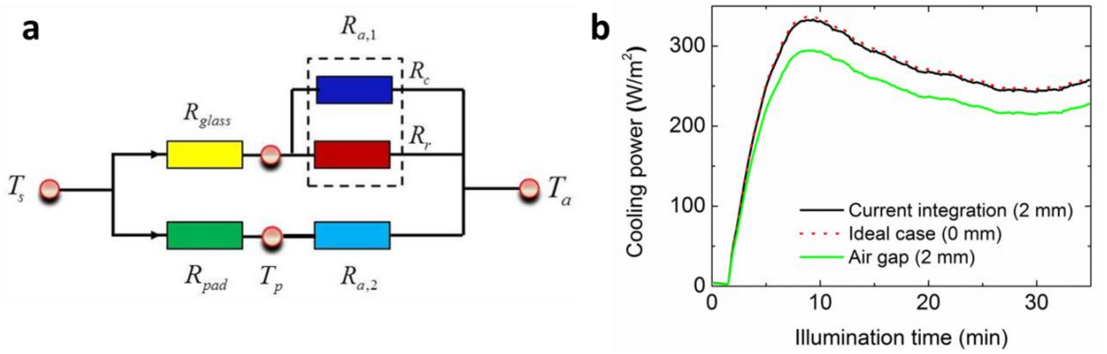

Figure 4. Thermal resistance network analysis. (a) The network for the analysis. (b) The cooling power of the device under different integrations. The accumulated heat partly flows through the glass layer (R_{glass}) and the combined resistance ($R_{a,1}$) considering the heat dissipation by natural convection (R_c) and thermal radiation (R_r);

while the other part of heat flows via the thermal pad (R_{pad}) and is dissipated to the ambient by the heat pipe plate ($R_{a,2}$). Thermal resistance of glass and thermal paste can be calculated according to the following equations

$$R_{glass} = \frac{\delta_{glass}}{\lambda_{glass} A_o} \tag{1a}$$

$$R_{pad} = \frac{\delta_{pad}}{\lambda_{pad} A_o} \tag{1b}$$

where δ_{glass} and λ_{glass} are the thickness and thermal conductivity of the glass on the solar cells, respectively; δ_{pad} and λ_{pad} represent the thickness and thermal conductivity of the thermal pad, respectively; A_o is the area of the solar cell. While thermal resistance caused by natural convection and thermal radiation is formulated as follow

$$R_c = \frac{1}{h_c A_o}, R_r = \frac{1}{\sigma_{sb} A_o \, (T_s + T_a)(T_s^2 + T_a^2)} \tag{2}$$

where h_c is convective heat transfer coefficient; σ_{sb} is the Stefan-Boltzmann constant; T_s and T_a represent the solar cell and the ambient temperature, respectively. Therefore, the total thermal resistance of different heat dissipation strategies is:

$$R_{a,1} = \frac{R_c R_r}{R_c + R_r} = \frac{1}{h_c A_o + \sigma_{sb} A_o (T_s + T_a)(T_s^2 + T_a^2)} \tag{3}$$

Based on the experimental conditions, we have:

$$R_{a,2} = \frac{1}{3} R_{a,1} \tag{4}$$

Consequently, the energy flow can be expressed as:

$$q_m \left(R_{pad} + \frac{1}{3} R_{a,1}\right) = [(1-\beta) q_s - q_m] \cdot (R_{glass} + R_{a,1}) \tag{5a}$$

$$q'_m \left(\frac{1}{3} R_{a,1}\right) = [(1-\beta) q_s - q'_m] \cdot (R_{glass} + R_{a,1}) \tag{5b}$$

where q_m and q'_m represent the heat removal flux by the heat pipe plate with thermal pad and under ideal condition (no thermal resistance between the solar cell and the heat pipe plate), respectively; q_s is the simulated solar irradiance and β denotes the electrical

efficiency of the solar cell. The relative error of the managed heat caused by the thermal resistance of the thermal paste is evaluated as:

$$\frac{q_m - q_m'}{q_m'} = \frac{-R_{pad}}{R_{glass} + \frac{4}{3}R_{a,1} + R_{pad}} \quad (6)$$

Based on the parameters of the solar cells (δ_{glass}= 3mm, λ_{glass}=0.5W/($m \cdot K$)) and the experimental conditions (T_a=20 °C, h_c=12.5$W/(m^2 \cdot K)$), the relative error for the current integration is 1.26%. It increases to 12.7% when there is an air gap of the same thickness (2mm).

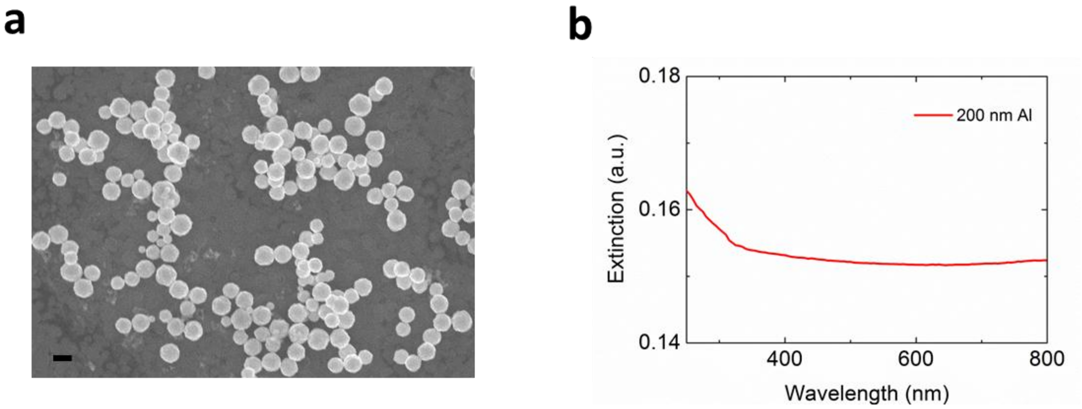

Figure 5. Al nanoparticle characterizations. (a) The scanning electron image (SEM) of the fabricated 200 nm Al nanoparticles (scale bar: 200 nm). (b) UV-Vis spectrum of the nanoparticles in DI water solution.

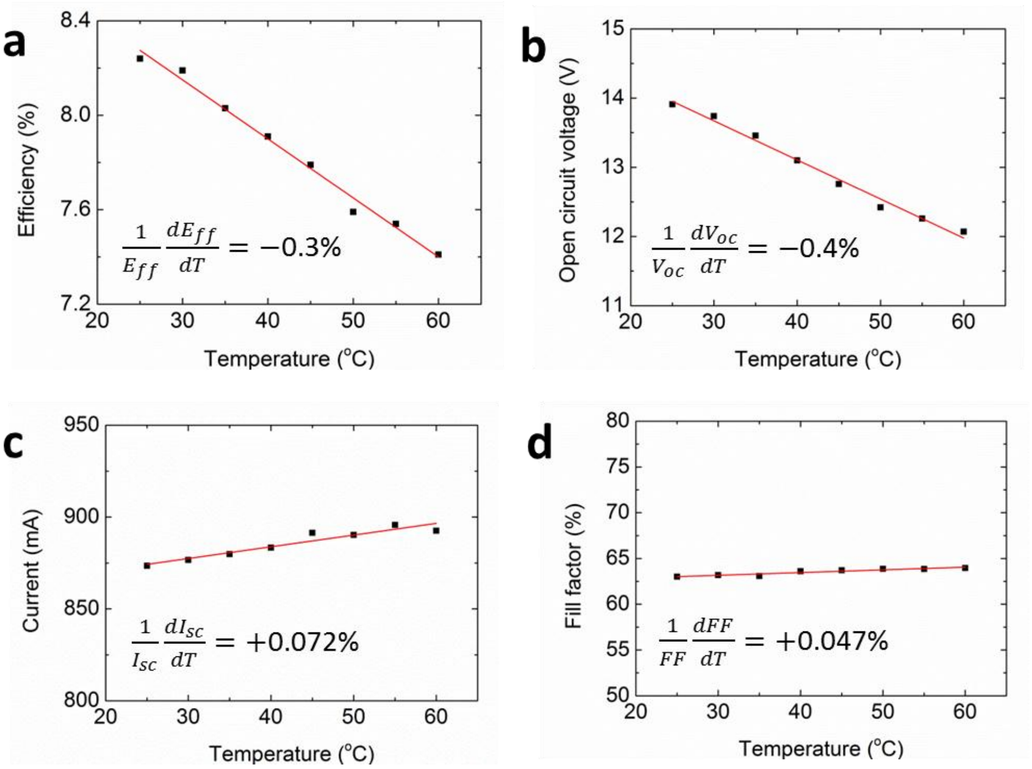

Figure 6. Temperature coefficients of the electrical parameters. (a) Efficiency, (b) open circuit voltage, (c) short circuit current and (d) fill factor as a function of the temperature for the investigated solar panels. The coefficients were obtained by linear fits of the experimental data. The coefficient of the efficiency is around -0.3%, which indicates approximately 10% efficiency reduction when the temperature increases by 35 ℃. The major factor that contributes to the efficiency reduction is the open circuit voltage V_{oc}, with a coefficient of -0.4%. The values of the current and the fill factor coefficients are both less than 0.1%, showing a slight increase.

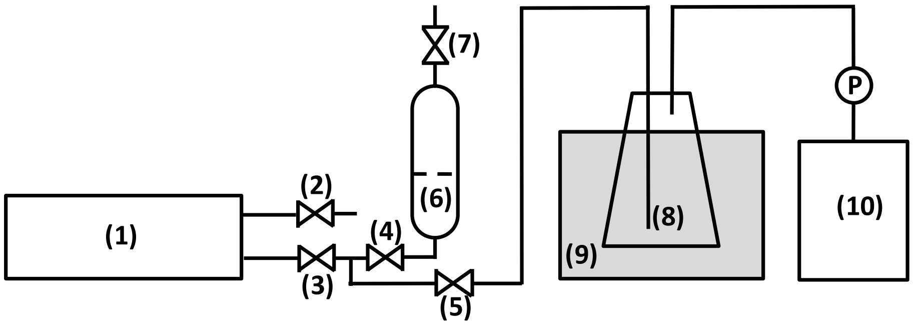

Figure 7. Apparatuses for charging the heat pipe. (1) heat pipe, (2) vacuum valve, (3) water valve, (4) release valve for the water flask, (5) vacuum valve, (6) flask containing degassed DI water (7) flask vacuum valve, (8) cold trap, (9) ice basket, (10) rotary vacuum pump. The procedures for charging are described as below.

- Step 1: Open valve (5) and evacuate the connecting tubing between valve (3) and valve (4), any water vapour will be trapped by the cold trap (8);
- Step 2: Close valve (5) to isolate vacuum pump from the system;
- Step 3: Open valve (4) completely to fill water into the connection tubing;
- Step 4: Open valve (3) so water can go into the panel, monitor the volume of water release (5 ml) by looking at the marks on the flask.
